# Climate Change‐Induced Landscape Alterations Increase Nutrient Sequestration and Cause Severe Oligotrophication of Subarctic Lakes

**DOI:** 10.1111/gcb.70314

**Published:** 2025-07-09

**Authors:** Willem Goedkoop, Sven Adler, Brian Huser, Hans Gardfjell, Danny C. P. Lau

**Affiliations:** ^1^ Department of Aquatic Sciences and Assessment Swedish University of Agricultural Sciences Uppsala Sweden; ^2^ Department of Forest Resource Management Swedish University of Agricultural Sciences Umeå Sweden

**Keywords:** Arctic, ecological change, NDVI, nitrogen, phosphorus, tundra, vegetation development

## Abstract

We combined decadal data (23–35 y) on nutrient concentrations for nine subarctic lakes with satellite imagery of vegetation (NDVI) to link the ongoing nutrient declines to the climate change‐induced greening of landscapes. Total phosphorus water concentrations (Total‐P) showed declining trends for all nine lakes, ranging from 1.5%–3.6%/y over the last decades. For most of the lakes' drainage areas, NDVI showed a dramatic increase during the 1990s and leveled off between 2001 and 2020. P sequestration in the lakes' drainage areas generally increased by 12%–30% between 1983–1994 and 2001–2020, with an exception of one high‐elevation lake for which P sequestration more than doubled. Area‐specific P‐sequestration estimates for 1983–1994 averaged 1.04 ± 0.10 tons P/km^2^ among all lakes but increased by 12%–33% for eight of the nine lakes during 2001–2020. Similar trends were found for nitrogen (N) sequestration, although these were an order of magnitude higher. These estimates illustrate long‐term changes in the sequestration of N and P by terrestrial vegetation in the region. Total‐P and DIN water concentrations showed negative correlations with both the NDVI_max_ of their drainage areas and plot‐scale measurements of tundra dwarf shrub cover. These correlations explained 51.8%–75.4% of the variability in declining nutrient water concentrations and showed the strong links between terrestrial vegetation development and declines in nutrient inputs to downstream lakes. Similar processes are likely ongoing in other parts of the Arctic where vegetation development is progressing, but are either not detected due to the lack of long‐term monitoring data or compensated for by nutrients released from thawing permafrost and/or thermokarst slumps. Upscaling our P‐ and N‐sequestration estimates for the nine lakes to the entire Arctic/alpine ecoregion in Sweden showed an average increase of 12.0 ± 1.7 Mtons P and 122.6 ± 17.5 Mtons N between the periods 1982–1994 and 2001–2020.

## Graphical abstract

- Warming of the Arctic regions stimulates plant growth and the storage of nutrients in plant biomass.
- Long‐term increases in plant biomass (greening) in the Swedish Arctic/alpine ecoregion were linked to the dramatic declines in nutrient concentrations in downstream lakes.
- Our calculations showed an average increase of phosphorus and nitrogen in plant biomass of 12%–33% between 1983–1994 and 2001–2020 for most lake drainage areas. For one high‐elevation lake, this increase exceeded 100%.

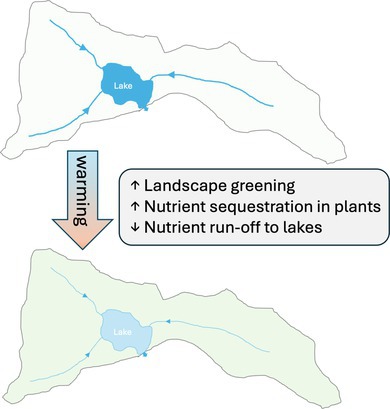

## 1 Introduction

The rate of global warming in the Arctic regions is more than three times higher than the global average (IPCC 2021; Rantanen et al. 2022), leading to permafrost thaws (Kokelj et al. 2013; AMAP 2021), shorter ice cover duration on lakes (Sharma et al. 2021), and increased vegetation development on land (Myers‐Smith et al. 2011; Elmendorf et al. 2012; Myers‐Smith and Hik 2018). Indeed, warming‐induced greening of the Arctic is currently a widely accepted concept (but see Myers‐Smith et al. 2020). Tundra vegetation development has been largely attributed to summer warming (Berner et al. 2020), especially so in Scandinavia (Hallinger et al. 2010; Myers‐Smith et al. 2015). There has, however, been a general lack of consensus regarding which climate variable is the main driver of tundra shrub growth, likely illustrating that the tundra is a heterogeneous biome (Myers‐Smith et al. 2015).

Also in northern Scandinavia, tundra vegetation is gradually being replaced by shrubs, mountain birch, and boreal forests, a process that has only just begun (Lagergren et al. 2024). Elmendorf et al. (2012) provided plot‐scale evidence of widespread tundra vegetation change across the Arctic, i.e., field‐level support for remote sensing observations that the Arctic biome is greening (Pouliot et al. 2009; Beck and Goetz 2011; Lagergren et al. 2024). Hedenås et al. (2016) used systematic inventory data to show that both total tree canopy, as well as tundra field vegetation (graminoids and dwarf shrubs) cover in the Arctic/alpine ecoregion of Sweden increased by 29% and 20%, respectively, between 2003 and 2012. Observed greening and vegetation development are a consequence of a longer growing season (Callaghan et al. 2004; Piao et al. 2019) and increased nutrient availability due to enhanced decomposition (Hartley et al. 1999; Sarneel et al. 2020) and weathering rates in soils (Thorn et al. 2001). Keenan and Riley (2018) showed that warming increased vegetation greenness in cold northern regions between 1982 and 2012, suggesting an expected decline in temperature limitation of plant communities under future warming scenarios. These terrestrial changes have important implications for downstream freshwater ecosystems (Wrona et al. 2016).

Concurrent with the greening of northern landscapes, lakes and rivers in boreal and subarctic regions are becoming more nutrient‐poor (Eimers et al. 2009; Arvola et al. 2011; Huser et al. 2018; Nilsson et al. 2024). For example, long‐term data have shown that Total‐P concentrations in lakes in Sweden have experienced successive declines (3%–4%/y) since the late 1980s (Huser et al. 2018), forcing many northern lakes toward ultra‐oligotrophic conditions with likely effects on community composition and biological production. While increases in dissolved organic carbon (DOC) concentrations have been observed in many boreal lakes (Eklöf et al. 2021 and references therein), subarctic and high‐elevation lakes generally have high water transparency and have shown no apparent increasing DOC trends (Isles et al. 2018, W. Goedkoop, unpublished data), likely due to the fact that their catchments have thin soils and sparse vegetation.

Dramatic declines in concentrations of key nutrients such as P and N, and subsequent oligotrophication, have obvious negative effects on the productivity of primary producers (i.e., algae, cyanobacteria) in lakes, both in pelagic (Isles et al. 2018; Bergström et al. 2020) and benthic habitats (Vadeboncoeur et al. 2001). Primary production and food webs in clearwater lakes are primarily driven by benthic primary production, which can occur down to much larger depths than in humic, boreal lakes (Devlin et al. 2013; Vander Zanden and Vadeboncoeur 2002; Vadeboncoeur and Power 2017). Cyanobacteria have competitive advantages over algae under nutrient‐poor conditions, as many species have the capacity to fix N_2_ (Diehl et al. 2018) and strategies to cope with seasonally low P concentrations (Adams et al. 2008; Sanz‐Luque et al. 2020). Nutrient‐induced shifts among benthic primary producers from algae to cyanobacteria may also influence the quality of basal resources, as algae, especially diatoms, are rich in polyunsaturated fatty acids that promote growth and development of consumers (Müller‐Navarra et al. 2000; Goedkoop et al. 2007) and trophic transfer efficiency (Brett and Müller‐Navarra 1997). Conversely, cyanobacteria lack these fatty acids (Napolitano 1999) and may produce highly potent neurotoxins (Christoffersen 1996; Metcalf and Codd 2014), making them a lower‐quality resource than algae. Such shifts in primary producer assemblages and overall primary production as a result of oligotrophication can thus have strong repercussions on the structural and functional biodiversity of northern and subarctic clearwater lakes.

In this study we quantified the sequestration of nutrients in vegetation in the drainage area of high‐latitude mountain lakes in Scandinavia and addressed the link between landscape greening and the ongoing oligotrophication of these lakes. We sampled tundra vegetation at multiple sites (3 plots/site) across a latitudinal gradient (62.2°–68.4°N) and analyzed samples for phosphorus (P) and nitrogen (N) concentrations. We then combined these data with Sentinel‐2 satellite imagery data to create relationships between the normalized difference vegetation index (NDVI) and plot‐scale nutrient concentrations of ambient vegetation. We used the resultant relationships to calculate decadal sequestration of P and N in the drainage areas of high‐latitude lakes and correlate these to the observed declines in lake water nutrient concentrations since the late 1980s. Lastly, we used vegetation data from National Inventories of Landscape in Sweden (NILS) to further quantify the link between increased vegetation coverage and nutrient declines in the study lakes. We hypothesized that increased nutrient retention rates in vegetation, both as NDVI and vegetation coverage, correlate with the long‐term declines in lake nutrient concentrations. We further hypothesized that the increase in sequestration rates of P and N in vegetation would be weaker in the northernmost area compared to the southern limit of the latitudinal gradient, because vegetation development likely is temperature dependent.

## 2 Materials and Methods

### 2.1 Lake Descriptions

**FIGURE 1:**
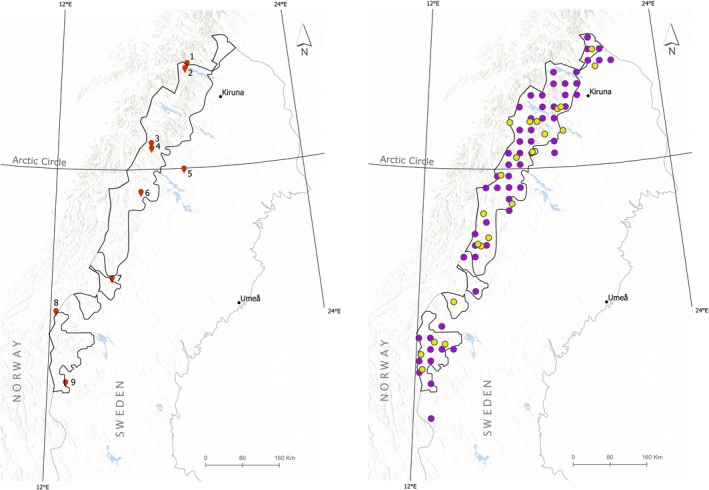
Maps of northern Scandinavia showing the locations of the study lakes and their catchments (left panel): 1 = Abiskojaure, 2 = Latnjajaure, 3 = Båtkajaure, 4 = Njalakjaure, 5 = Louvvajaure, 6 = Stor Tjulträsk, 7 = Dunnervattnet, 8 = Stor Björsjön, 9 = Övre Fjätsjön, and of the 59 NILS monitoring sites (purple) and 22 NILS‐Alpine sites (yellow) where vegetation samples were collected for elemental analysis (right panel). The black contour delineates the Arctic/alpine ecoregion in Sweden, and three meridians are shown to facilitate global orientation. For further details see text.

The nine study lakes are situated at elevations exceeding 400 m on the eastern slopes of the Scandes Mountain Range (Figure 1, hereafter referred to as The Scandes), the mountain divide that is part of the Arctic and alpine ecoregion in Scandinavia (Nordic Council of Ministers 1984; Gustafsson and Ahlén 1996). These lakes have been part of the Swedish National Monitoring Program since the late 1980s or mid‐1990s, with monitoring conducted by the same accredited laboratory (SWEDAC, Swedish Board for Accreditation and Conformity Assessment) over this period. Alkalinity, pH, major ions, and nutrients have been measured consistently over time using standardized methods (see Fölster et al. 2014 for a detailed description of the Monitoring Program). Here we use epilimnetic water samples (0.5–1.0 m water depth) collected three to four times per year, i.e., spring, summer, fall, and winter. Morphometric and catchment size data, as well as long‐term mean water concentrations of total phosphorus (Total‐P), dissolved inorganic nitrogen (DIN), and total organic carbon (TOC) are shown in Table 1. Catchment size and land cover for the study lakes are given in Table 2.

**TABLE 1:** Morphometric and catchment size data, as well as long‐term (1988/1996–2022) annual mean water concentrations of total phosphorus (Total‐P), dissolved inorganic nitrogen (DIN), and total organic carbon (TOC).

Lake	Latitude (dec)	Longitude (dec)	Elevation (m a.s.l.)	Area (km^2^)	D_mean_ (m)	Catchment (km^2^)	Total‐P (μg/L)	DIN (μg/L	TOC (mg/L)
Latnjajaure	68.3506	18.4883	976	0.74	16.5	9.46	2.7 (0.5–7.3)	25 [6.6–58)	1.0 (0.5–5.2)
Abiskojaure	68.4453	18.6139	487	2.79	12.8	366.9	4.5 (1.8–9.6)	32 (15–73)	1.6 (1.0–5.1)
Båtkåjaure	66.9145	16.6113	631	0.63	4.2*	4.76	2.5 (1.2–6.5)	14 (5.5–38)	2.6 (1.9–5.7)
Njalakjaure	66.8169	16.6221	849	0.33	5.8*	4.28	3.1 (1.4–9.0)	19 (5.0–33)	1.5 (0.7–4.8)
Louvvajaure	66.3931	18.1695	456	0.82	4.9*	4.16	3.6 (1.7–10.3)	18 (6.8–42)	3.5 (2.5–7.2)
Stor Tjulträsket	65.9625	16.0554	532	5.25	21.2	277.4	4.8 (2.3–10.4)	43 (21–86)	2.3 (1.3–3.8)
Dunnervattnet	64.2835	14.6917	445	2.67	5.8*	100.8	4.0 (2.3–6.0)	28 (13–110)	5.9 (4.7–8.1)
Stor Björsjön	63.6151	12.2426	561	0.33	4.7	20.54	4.0 (2.5–9.5)	22 (8.8–41)	4.8 (3.8–6.1)
Övre Fjätsjön	62.2359	12.7684	743	0.91	4.2	43.19	8.3 (3.8–26.7)	41 (17–84)	4.4 (3.4–7.6)

*Note:* Concentrations are given as mean annual values (based on 3–5 monthly surface water values) and the range for the period 1988/1996–2022. D_mean_ = Mean depth. Note that mean depths marked with an asterisk were calculated according to Sobek et al. (2011).

**TABLE 2:** Catchment size and land cover for the nine study lakes.

Lake	Catch‐ment (km^2^)	Water surface (km^2^)	Forest (km^2^)	Wetland (km^2^)	Bare rock (km^2^)	Tundra veg. (km^2^)	Developed land (km^2^)	Other (km^2^)
Latnjajaure	9.46	0.77	0	0.01	7.09	1.58	0	0
Abiskojaure	366.9	15.02	18.61	2.19	117.40	193.95	0	0
Båtkåjaure	4.76	0.75	0.38	0.18	0.18	3.27	0	0
Njalakjaure	4.28	0.36	0	0.03	3.65	0.25	0	0
Louvvajaure	4.16	0.80	2.40	0.09	0	0.34	0.04	0.48
Stor Tjulträsket	277.4	14.99	52.26	16.50	63.45	129.67	0.11	0.37
Dunnervattnet	100.8	7.89	58.39	14.97	0.84	15.46	0.37	2.87
Stor Björsjön	20.54	0.88	2.04	5.13	0	7.76	0.04	0.01
Övre Fjätsjön	43.19	2.23	6.59	5.38	1.00	27.91	0.03	0

*Note:* Forest mainly refers to mountain birch forest, but with an increasing share of pine and spruce at lower elevations and latitudes. Tundra vegetation (Tundra veg.) refers to dwarf shrubs (i.e., species belonging to Vaccinium, Cassiope, Kalmia, Empetrum, Phyllodoce, Rhododendron, Calluna, Arctostaphylos, Harrimanella, Arctous, Andromeda). Note that the sum of land cover does not sum up to 100% for Abiskojaure and Stor Björsjön, as part of their catchments, i.e., 19.72 km^2^ (or 5.4%) and 4.68 km^2^ (or 22.8%), respectively, are located in Norway.

The Arctic/alpine ecoregion traverses the Arctic circle in northern Scandinavia and is part of the global subarctic region, but its high elevation contributes to the Arctic temperature regimen even at relatively low latitudes (i.e., between 62° and 69° north). Mean annual temperatures in the ecoregion range from 0°C in the south to −3°C in the north, the growth period is < 140 d/y, and permafrost is patchy/discontinuous (Brown et al. 2000). The ecoregion is covered by mountain birch forest ( *Betula pubescens* var. *czerepanovii*), shrubs (*Salix* spp) at low and intermediate elevation, and dwarf shrubs (i.e., species belonging to *Vaccinium, Cassiope, Kalmia, Empetrum, Phyllodoce, Rhododendron, Calluna, Arctostaphylos, Harrimanella, Arctous, Andromeda*) vegetation and bare rock above the treeline. The treeline is currently at 700–1000 m but has been expanding markedly during the last century in response to warming (Kullman 2010, 2018). The region has experienced continuous warming since the 1980s with highly significant increases in daily air temperatures, i.e., the June–September monthly average has increased by 0.038°C ± 0.005°C/y between 1985 and 2020 across our study lakes (Figure S1). The predominant land‐use is reindeer herding by Indigenous Sami people, who use the high‐elevation areas for reindeer grazing in summer. Otherwise the mountain range is mostly used for hiking and fishing, while ski resorts cause limited local stress on ecosystems. The remote study lakes are not affected by hydropower and receive negligible point source pollution. Lake size and catchment size of the study lakes ranged from 0.33–5.25 km^2^ and 4.06–370 km^2^, respectively (Table 1).

### 2.2 Vegetation Monitoring Sites and Sample Collection

The Swedish alpine vegetation has been monitored from 2003 to 2020 at 59 monitoring sites (5 km × 5 km) above the treeline along the Scandes as part of the NILS vegetation monitoring program (Ståhl et al. 2011). The monitoring sites are split into five annually sampled subsets, which all comprise evenly distributed quadrats over the alpine region (Figure 1). Thus, one fifth of the total sample size is inventoried each year, and each monitoring site is revisited after 5 years (see Ståhl et al. 2011 for details). Within each NILS site, 12 sample plots are regularly placed. In contrast to Hedenås et al. (2016), we analyzed changes in ground and field layer vegetation coverage of three small 0.28‐m radius subplots (0.25 m^2^) instead of the whole 10‐m radius sample plot in order to minimize observer variation. These three 0.25‐m^2^ plots were placed just outside the larger plots and had similar vegetation (as judged from field images). The coverage estimation was done by using a simple Horvitz–Thompson estimator (Horvitz and Thompson 1952) and calculated with the survey library (Lumley 2004). Using these data, we computed a 5‐y moving average of the mean for dwarf shrub cover estimates for our analysis.

In 2021 the design of NILS was modified and got a more representative (225 monitoring sites, 1 km^2^) and effective two‐step, balanced sampling design in which each site, consisting of 12 sampling plots (radius 10 m), was visited every 5th year. Within half of the sites visited in 2021, we selected two sample plots of twelve for biomass collection in three subplots (see below). These plots were chosen as they are predominated by tundra vegetation, i.e., dwarf shrubs and grasses. In order to avoid removing vegetation within the 10‐m radius plots used for long‐term vegetation monitoring, three smaller plots (area 0.25 m^2^) were placed just outside of these 10‐m plots (north, south‐east, south‐west, i.e., a 120° angle between them) and marked with a ring. Within this ring surface, the total ground and field layer plant coverage, the mean height of vegetation, and the coverage of bare soils/rock were estimated.

The plots were then photographed before and after above‐ground vegetation was removed quantitatively using a scissor. Collected plant biomass from each of 38 sites (*n* = 3 plots per site) was air‐dried, transferred to marked paper bags, and packed for transport to the laboratory. The samples were stored at 4°C in the dark and then processed and analyzed for N and P. For this, samples were dried (65°C, 24 h) and weighed to determine dry mass, homogenized using a blender, and stored in desiccators. Subsamples were analyzed for N using a LECO TruMac elemental analyzer. QA/QC for these analyses included two reference materials (a commercially available wheat standard and an internally produced standard soil). Internal standards of these materials were run with every batch (40 samples) to guarantee analytical accuracy (i.e., deviation was < 3%). Also an internal standard was also analyzed (every 10th sample) to check for instrumental performance. Total‐P was analyzed using an ICP‐Avio 200 after digestion with concentrated HNO_3_ (SS‐28311 2017).

### 2.3 Catchments, NDVI, Water Chemistry, and Temperature Data

The NILS‐monitoring sites, with their good coverage of sites across the Arctic/alpine ecoregion in Sweden, provide information on vegetation change at the landscape level (Figure 1). The catchments of the study lakes are all embedded within this landscape. Catchments were delineated by snapping the sample site coordinates to the lake outlet and calculating the watersheds upstream from directional d8 rasters that are based on a modified 10‐m resolution digital elevation model (DEM10) of Sweden supplied by the Swedish Mapping, Cadastral and Land Registration authority. The DEM10 is a resampling of a 2‐m resolution DEM augmented with the national map of hydrography, available from the Swedish Meteorological and Hydrological Institute. This was done using a python script (vivan2) using tools from ArcGIS python library arcpy. Drainage areas were calculated by subtracting the lake area from the catchment size.

NDVI data for the drainage areas of our study lakes (i.e., catchment area minus area of the study lake) were extracted from the NOAA Climate data record (NOAA 2023, normalized NDVI version 4, Vermote et al. 2014) using Google Earth engine (Gorelick et al. 2017) through the R package rgee (Aybar 2023). NDVI quantifies vegetation biomass by calculating the differences between near‐infrared (which vegetation reflects) and red light (which vegetation absorbs) divided by their sum. For each catchment, the maximum NDVI value > 0.2 for the snow‐free period of the year was extracted for each year and the mean of NDVI_max_ over the whole drainage area was calculated. NDVI values taken before 7:00 am were filtered out. By doing so we could compile NDVI_max_ data back to 1983. Annual peak NDVI (or NDVI_max_) have shown good correlation with total aboveground phytomass in earlier studies (Shippert et al. 1995; Walker et al. 2003).

For each of the 38 NILS sample plots where plant biomass was collected in 2021 (see above), the NDVI_max_ values for 2021 were derived from Sentinel‐2 scenes (10 m × 10 m resolution). As each NILS sample plot of 314 m^2^ covered 4–8 Sentinel pixels, we first calculated the mean NDVI value of these pixels and then selected the maximum NDVI for the snow‐free period for each plot (i.e., NDVI_max_). The procedure is summarized in Figure S2. We then carefully scrutinized the photos of the vegetation sites and omitted five plots where not all vegetation could be removed and another four plots that were partly snow‐covered, resulting in a data set covering 29 sites. As a next step, relationships were established between NDVI_max_ of NILS monitoring plots and the concentrations of P and N of the tundra vegetation samples collected there.

NDVI data were then recalculated into units of P and N using established relationships between NDVI_max_ and the concentrations of P and N in collected tundra vegetation samples from the NILS sites. These calculations provided an estimate of vegetation development in the drainage areas (i.e., catchment size excluding water surfaces) of the study lakes over the time frame of the study (i.e., 1983–2020). P‐ and N‐sequestration by vegetation in the drainage areas for most lakes was calculated for the periods of 1983–1994 and 2001–2020, but for Abiskojaure we compared sequestration for the periods of 1983–1992 with those for 2001–2020 as NDVI increased markedly already in 1993. These data were then upscaled to the entire Arctic/alpine ecoregion (i.e., 46,528 km^2^) by using the mean (±SE) of these nine drainage areas. In addition, we used a high‐resolution map of tundra vegetation from the Swedish Mapping, Cadastral and Land Registration Authority in order to upscale our P‐ and N‐sequestration data to the tundra part (i.e., 33,935 km^2^ or 73%) of the Arctic/alpine ecoregion in Sweden.

Water chemistry data for the lakes were extracted from databases for national freshwater monitoring (see above) available at the Swedish University of Agricultural Sciences (SLU). Swedish or European standards have been used for spectrometric analyses of Total‐P (SS‐EN ISO 6878:^2005^), NH_4_‐N (SS‐EN ISO 15923‐1:^2024^), and NO_2_+NO_3_‐N (SS‐EN ISO 13395:^2005^). Because the lab has changed analytical methods for Total‐N several times, resulting in inconsistent data series, we chose to use the sum of concentrations of dissolved inorganic nitrogen (DIN, i.e., sum of NO_2_‐N, NO_3_‐N and NH_4_‐N) to analyze long‐term changes in nitrogen concentrations. DIN has shown to be a good indicator of nitrogen availability for phytoplankton production in northern lakes (Lau et al. 2021), while the DIN/Total‐P ration has been used as an indicator for nutrient limitation regimes of phytoplankton in northern lakes (Bergström 2010). Annual mean values based on 3 or 4 monthly sampling occasions were used in time series analysis.

Air temperature data were obtained from the Climate Research Unit Time‐series database CRU TS version 4.06 (Harris et al. 2020). Lake coordinates were used to extract daily mean temperatures on 0.5° latitude × 0.5° longitude grids for the months June, July, August, and September of 1985–2020. These months reflect the annual growth season, i.e., the months in which no temperatures below 0°C were recorded.

### 2.4 Statistical Analysis

Generalized Additive Mixed Effect Models (Hastie and Tibshirani 1990) were used to analyze relationships between lake nutrient water concentrations (i.e., Total‐P and DIN) and NDVI_max_ and dwarf shrub coverage monitoring data, respectively, using R (R Core Team 2023), including the packages mgcv (Wood 2011), dplyr (Wickham et al. 2023), terra (Hijmans 2024), sf (Pebesma and Bivand 2023) and ggplot2 (Wickham 2016). These models analyzed the overall relationships between landscape greening and lake oligotrophication. Time series analyses of nutrient water concentrations and NDVI_max_ were done in R, using mixed‐generalized mixed models (GAM) smooth fits with lake as a random factor and year as a predictor, to visualize trends for each of the study lakes. Mann–Kendall tests were then used for testing these temporal trends for their significance. Student *t*‐tests, to compare nutrient sequestration between time periods, and other statistics were done using JMP (JMP Pro 15 2019) with alpha set at 0.05.

## 3 Results

**FIGURE 2:**
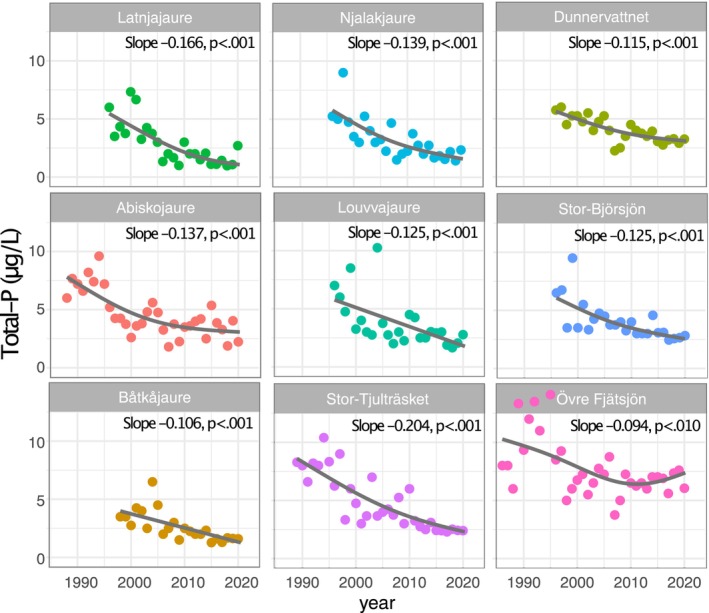
Temporal trends in annual mean total phosphorus water concentrations (Total‐P) for the nine study lakes. Line fits show the result of a simple GAM smooth fit. Sen's slopes and *p*‐values (Mann–Kendall) are given in the panels.

**FIGURE 3:**
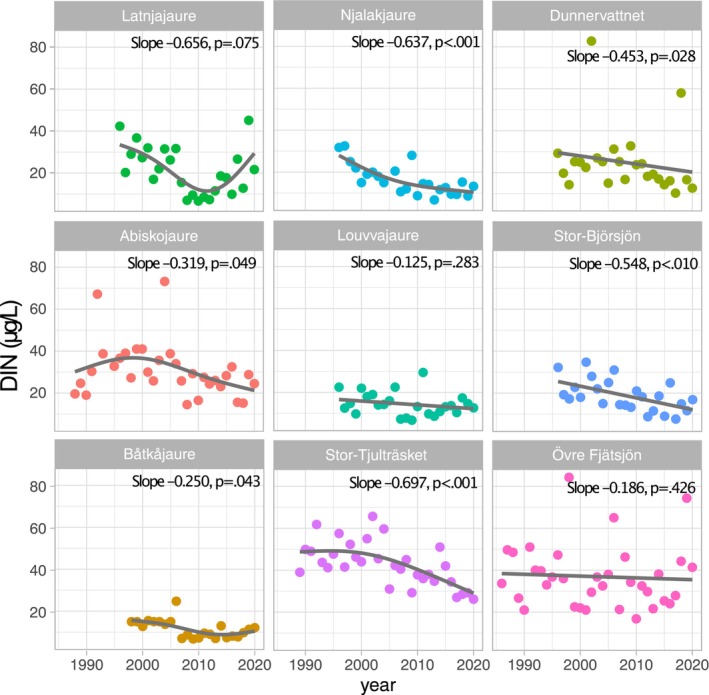
Temporal trends in mean annual dissolved inorganic nitrogen water concentrations (DIN) for nine study lakes. Line fits show the result of a simple GAM smooth fit. Sen's slopes and *p*‐values (Mann–Kendall) are given in the panels.

Total‐P water concentrations showed highly significant declining trends for all nine lakes (Figure 2), ranging from 1.5%/y (Övre Fjätsjön, 1986–2020) to 3.6%/y (Njalakjaure, 1996–2020) over the last few decades. For example, mean Total‐P in Njalakjaure declined from 5.3 to 1.2 μg/L between 1996 and 2020 (or 3.1%/y), while the much larger lake Stor‐Tjulträsket showed a similar decline, i.e., from 8.5 to 1.8 μg/L between 1989 and 2020 (or 2.5%/y). All lakes except for the southernmost Övre Fjätsjön had Total‐P concentrations that declined to values close to or below 3 μg/L for the latest years in the data set. DIN‐water concentrations showed negative slopes for all lakes, ranging from −0.125 (Louvvajaure) to −0.697 (Latnjajaure). The declining DIN‐trends were significant for six of nine lakes, i.e., Abiskojaure, Båtkåjaure, Njalakjaure, Dunnervattnet, Stor‐Björsjön, and Stor‐Tjulträsket, and borderline significant (*p* = 0.075) for Latnjajaure (Figure 3). These declining trends in nutrient concentration illustrate the severe oligotrophication of lakes in the region.

**FIGURE 4:**
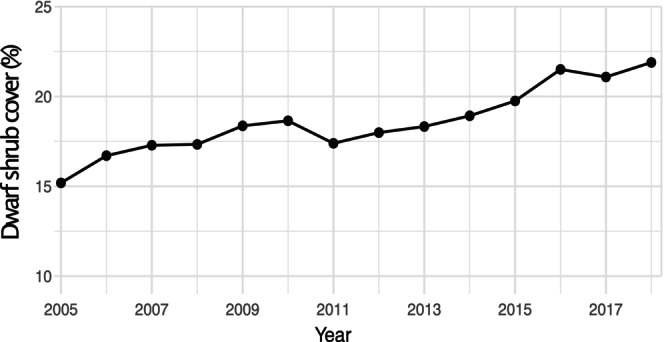
Temporal trend in dwarf shrub vegetation cover from the NILS‐alpine monitoring program collected during 2003–2020. Data points show a five‐year moving average based on 507 observational plots of tundra vegetation at 59 monitoring sites (see Figure 1). Dwarf shrubs include species belonging to Vaccinium, Cassiope, Kalmia, Empetrum, Phyllodoce, Rhododendron, Calluna, Arctostaphylos, Harrimanella, Arctous, and Andromeda.

Time series of the NDVI_max_ for the lake catchments showed instead strong positive trends from the 1980s (Figure S3), demonstrating a gradual, warming‐induced increase in vegetation development. For most of the lakes' drainage areas, NDVI_max_ increased dramatically already during the 1990s, but then leveled off between 2001 and 2020. The only lake that showed an opposite trend was Båtkajaure, a high‐elevation lake that instead showed an increase in NDVI_max_ during later years in the data set. These results were supported by plot‐level measurements of dwarf shrub coverage that showed a 7% increase (i.e., from 15% to 22%) on tundra sites during 2003–2020 from the NILS‐monitoring program (Figure 4).

**FIGURE 5:**
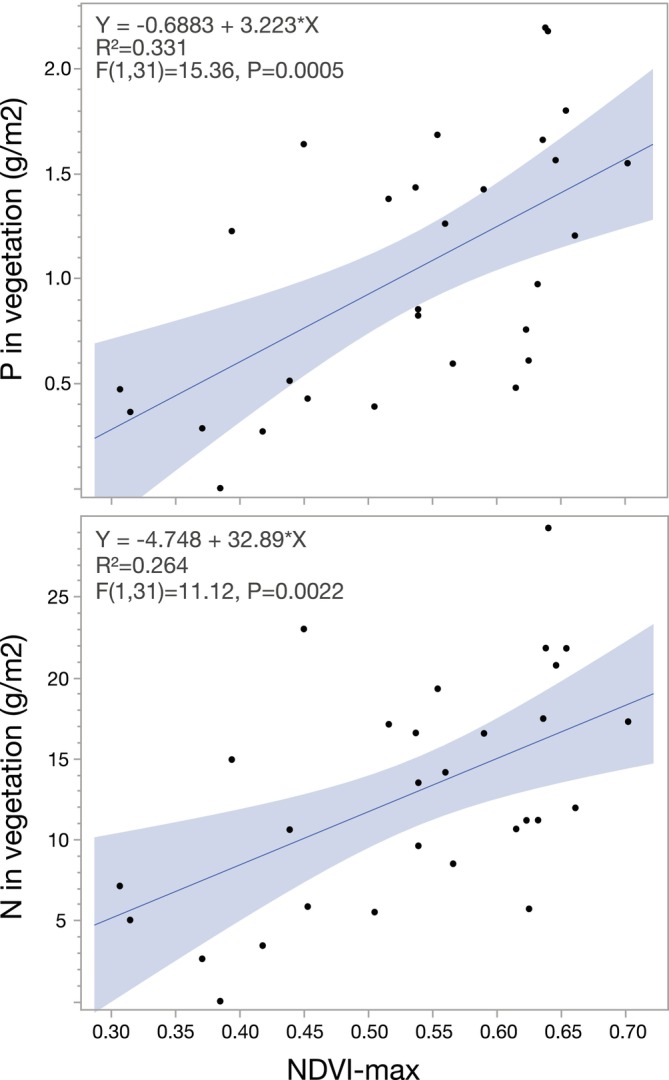
Relationships between annual maximum NDVI (NDVI_max_) and the concentration of P (upper panel) and N (lower panel) in plot scale (Sentinel, 10 × 10 m) tundra vegetation at 33 sites (*n* = 3 per site) in the Scandes. Linear relationships and their statistical details are given in the top left corner of the panels. For more details see text.

**FIGURE 6:**
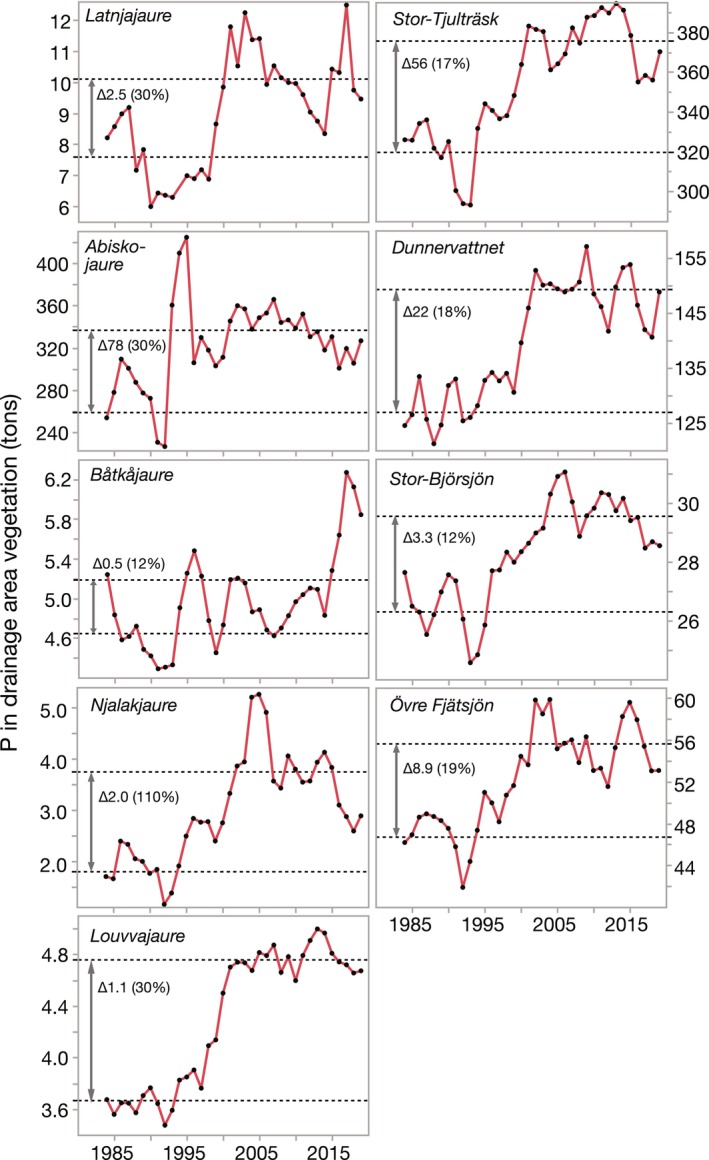
Time trends (1983–2020) of phosphorus (P) in drainage area vegetation for nine Arctic/alpine lakes. The red lines show the three‐year moving average, while dotted lines show the means for the periods 1983–1994 (NB 1983–1992 for Abiskojaure) and 2001–2020, respectively. Adjacent to the double‐sided arrow is the absolute and relative difference between the two intervals. Note the differences in scale for the Y‐axes.

**FIGURE 7:**
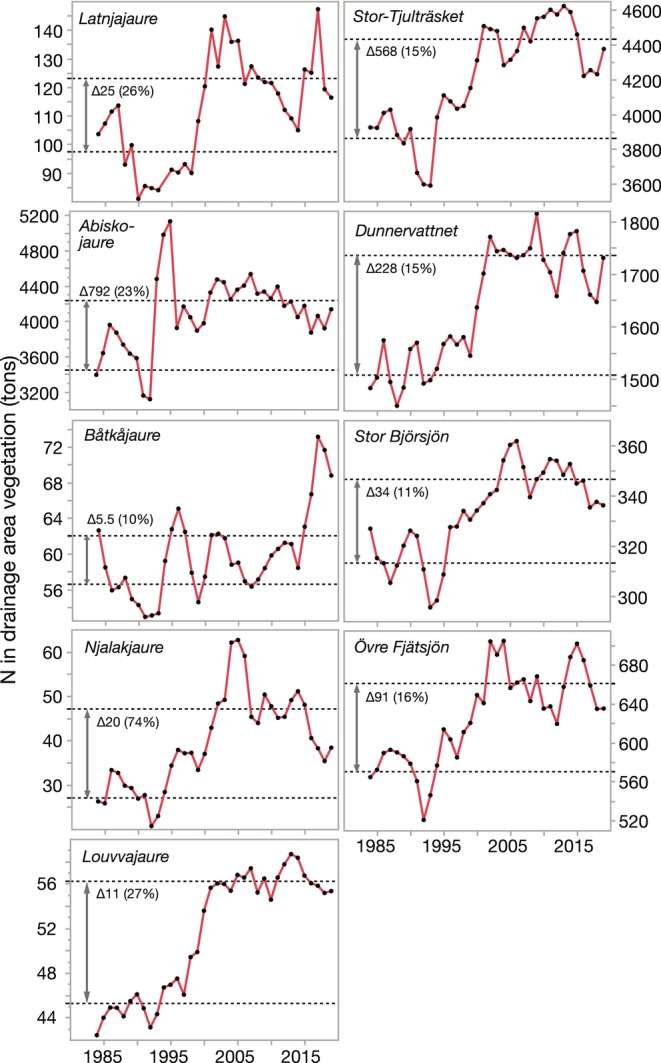
Time trends (1983–2020) of nitrogen (N) in drainage area vegetation for nine Arctic/alpine lakes. The red lines show the three‐year moving average, while dotted lines show the means for the periods 1983–1994 (NB 1983–1992 for Abiskojaure) and 2001–2020, respectively. Adjacent to the double‐sided arrow is the absolute and relative difference between the two intervals. Note the differences in scale for the Y‐axes.

Concentrations of P and N in vegetation samples showed highly significant linear relationships with plot‐scale NDVI_max_ (Figure 5). These relationships between the NDVI_max_ and nutrient concentrations of tundra plot vegetation were then used to calculate the overall temporal trends in P‐ and N‐sequestration by vegetation in the drainage area of the study lakes and the area‐specific sequestration for the two selected time periods. This upscaling from plot‐scale to the drainage area showed that the rapid increase in NDVI_max_ during the 1990s translated into marked increases in annual sequestration rates of P in drainage area vegetation, which then leveled off after the turn of the century for most lakes (Figure 6). P sequestration generally increased by 12%–30% between the time periods 1983–1994 and 2001–2020, with the exception of the high‐elevation Njalakjaure that more than doubled P sequestration in its small drainage area (3.92 km^2^). The total increase in sequestered P between the two periods ranged between 0.54 (Båtkajaure) and 77.6 tons (Abiskojaure) among the nine lake drainage areas. Trends for N‐sequestration in the drainage area were similar to those for P, with increases between 1983–1994 and 2001–2020 ranging from 10% to 27% among eight of nine lakes, but substantially higher for Njalakjaure at 74% (Figure 7). The increase in sequestered N between the two periods ranged from 5.5 tons (Båtkajaure) to 792 tons (Abiskojaure) among the nine lakes drainage areas.

Area‐specific P‐sequestration estimates for 1983–1994 averaged 1.04 ± 0.10 tons P/km^2^ among lakes and ranged from 0.46 ± 0.07 (Njalakjaure) to 1.37 ± 0.03 tons P/km^2^ (Dunnervattnet), but then increased by 12%–33% for eight of nine lakes during the period of 2001–2020, whereas the increase in sequestration for Njalakjaure was 110% (Table 3). Area‐specific N‐sequestration averaged 12.91 ± 1.02 tons P/km^2^ during 1983–1994 and 15.54 ± 0.78 tons N/km^2^ during 2001–2020, implying relative increases of 10%–74%. These estimates illustrate the long‐term sequestration of N and P by terrestrial vegetation in the region. Upscaling our P‐ and N‐sequestration estimates, using the mean (±SE) area‐specific differences in sequestration rates between 1982–1994 and 2001–2020 for the drainage areas of the nine study lakes, resulted in an increase of 12.0 ± 1.7 Mtons P and 122.6 ± 17.5 Mtons N being sequestered across the entire Swedish Arctic/alpine ecoregion. Similar values for only the share of tundra vegetation in this ecoregion (which was 73% of the total area) were 8.8 ± 1.2 Mtons P and 89.4 ± 12.7 Mtons N. Considering that most of the increase occurred over a time interval of 22.5 years (i.e., between the mid‐interval years) implies annual sequestration rates in above‐ground biomass of tundra vegetation of 390 tons P/y and 3973 tons N/y.

**TABLE 3:** Area‐specific sequestration rates of P and N (as ton/km^2^) stored in drainage area vegetation (mean ± 1 standard deviation) of nine Arctic/alpine lakes for time periods 1983–1994 and 2001–2020, as well as the relative increase (∆%).

Lake	P (ton/km^2^)			N (ton/km^2^)		
	1982–1994	2001–2020	∆%	1982–1994	2001–2020	∆%
Latnjajaure	0.88 ± 0.09	1.17 ± 0.06	33**	11.24 ± 0.86	14.17 ± 0.58	26**
Abiskojaure	0.74 ± 0.06^1^	0.96 ± 0.02	30**	9.80 ± 0.63^1^	12.05 ± 0.20	23**
Båtkåjaure	1.16 ± 0.05	1.30 ± 0.04	12*	14.13 ± 0.55	15.51 ± 0.35	10*
Njalakjaure	0.46 ± 0.07	0.96 ± 0.07	110***	6.93 ± 0.71	12.05 ± 0.75	74***
Louvvajaure	1.07 ± 0.03	1.42 ± 0.02	32***	13.22 ± 0.26	16.75 ± 0.15	27***
Stor‐Tjulträsket	1.22 ± 0.05	1.43 ± 0.02	17***	14.73 ± 0.48	16.89 ± 0.18	15***
Dunnervattnet	1.37 ± 0.03	1.61 ± 0.02	18*	16.23 ± 0.32	18.68 ± 0.20	15***
Stor Björsjön	1.34 ± 0.03	1.50 ± 0.02	12***	15.95 ± 0.26	17.63 ± 0.16	11***
Övre Fjätsjön	1.14 ± 0.03	1.36 ± 0.03	19***	13.93 ± 0.30	16.14 ± 0.29	16***
Average	1.04 ± 0.10	1.30 ± 0.07		12.91 ± 1.02	15.54 ± 0.78	

*Note:* Asterisks show the level of significance for *t*‐tests (*0.05 > *p* > 0.01, **0.01 > *p* > 0.001; ***p < 0.001). Lakes ordered north to south.

^1^ 1983–1992. See text.

**FIGURE 8:**
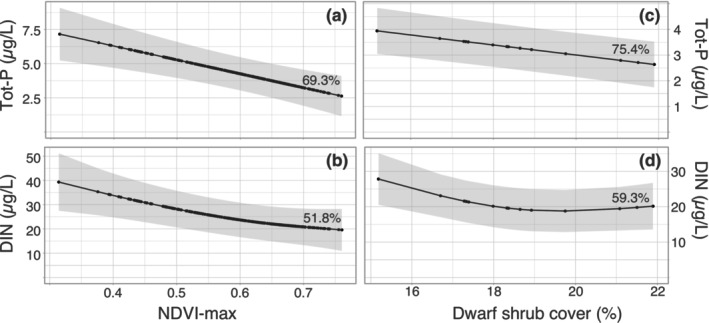
Generalized Additive Mixed Effect Model (GAM) with nine lakes as random factors for drainage area NDVI_max_ vs. Total‐P (a) and DIN water concentrations for the period 1988/1996–2020 (b), as well as for dwarf shrub cover (5‐y moving average, 2003–2020) vs. Total‐P (c) and DIN (d) water concentrations (intervals as above). Numbers adjacent to the line fits give the deviance explained by the model. The grey fields show the 95% confidence limit (*p* ≤ 0.009 for all).

Both Total‐P and DIN water concentrations for the nine lakes showed strong, negative correlations with the NDVI_max_ values of their drainage areas, which explained 69.3% and 51.8% of the variability, respectively (Figure 8A,B). Similarly, significant negative correlations were observed between Total‐P and DIN water concentrations and plot‐scale measurements of dwarf shrub coverage, which explained 75.4% and 59.3% of the variability in nutrient concentrations, respectively (Figure 8C,D). These relationships show the strong links between two measures of vegetation development in the drainage area and concurrent declines in nutrient concentrations in downstream lakes.

## 4 Discussion

Our study provides unique estimates of N and P sequestered by expanding vegetation in the drainage areas of subarctic lakes. This evidence demonstrates that temporal trends in catchment‐scale greening (as NDVI) and terrestrial vegetation development (as plot‐scale coverage) are closely associated with the ongoing, dramatic oligotrophication of lakes (as nutrient declines). Both the marked increases in NDVI_max_ and aboveground dwarf shrub coverage data explained a significant share of the decline in lake Total‐P and DIN concentrations (Figure 8). Moreover, these findings support experimental findings by Aerts et al. (2006) and that climate warming impacts on both the phenology and performance of plants from the cold biomes. Indeed, shrubification and tree line expansion are progressing also in the Scandes and have accelerated when the unusually cold 1980s were followed by markedly warmer decades and substantially longer vegetation periods (SMHI 2024; Kullman 2021a). Observed nutrient sequestration rates increased rapidly during the warm years of the 1990s (Figures 4 and 5), and coincided with the period of most dramatic declines in Total‐P water concentrations in the study lakes (Figure 2). These comparisons suggest that a number of exceptionally warm years apparently were a trigger for long‐lasting, positive effects on the establishment and growth of plants at higher elevations, which was the main driver of the concurrent lake oligotrophication.

Similar vegetation development is ongoing elsewhere in the Arctic but the association between terrestrial sequestration of P and N and lake oligotrophication is either not detected due to the lack of long‐term monitoring data or the fact that changes were masked by nutrients released during large‐scale permafrost thaws (Reyes and Lougheed 2015) or regional thermokarst slumps (Kokelj et al. 2013). In the subarctic region of northern Scandinavia, where permafrost is patchy and discontinuous and such permafrost‐derived nutrient subsidies are likely marginal, this is reflected by the absence of increasing DOC trends for our high‐latitude study lakes. Our large‐scale results show that any positive effects of warming on nutrient availability are indeed entirely counteracted by an increased uptake by terrestrial vegetation.

The low NDVI_max_ values early in the time series can be attributed to the unusually cold 1980s, when soil temperatures in the mid‐range of the Scandes were 3°C–5°C lower (and around zero) than during the 1990s (Kullman 2021a). During the 1980s, germinability of tree species was suppressed and snow fields over the summer were more common (Kullman 2002). During the warmer 1990s, the joint effects of snow cover decline (Kullman 2002; Callaghan et al. 2011), increased tundra vegetation development (Figure 3, but see also Hedenås et al. 2016), and the progression of mountain birch forest stands to higher elevations (Kullman 2021b; Nygaard et al. 2022) have contributed to an increased greening of the region. This expansion of vegetation is associated with the observed gradual increase in NDVI_max_ during the recent warmer decades compared with the colder period from 1983–1994 (Figure S1). Using data from 1740 long‐term vegetation monitoring plots, Hedenås et al. (2016) reported that the extension of birch forest cover in the Swedish Scandes was similar between the periods 2003–2007 and 2008–2012, but total canopy cover increased only during 2008–2012. Similarly, Hedenås et al. (2016) found that the coverage of graminoids and dwarf shrubs, as well as the total vegetation cover increased in both tundra and the understory in birch forest stands. These observations support our finding that NDVI_max_ increased until approximately 2010, and was then followed by a leveling off in most catchments (Figure S3). These findings are in concordance with Berner et al. (2020), who showed that summer warming is the main driver of widespread greening of the Arctic tundra and that effects are stronger in the Low Arctic and Oro Arctic (i.e., alpine, tundra), to which northern Scandinavia belongs.

Our estimates of nutrient sequestration, however, have potential inherent errors as relationships established for tundra vegetation (Figure 5) were used to extrapolate to entire drainage areas that also contain slowly extending mountain birch and to a lesser extent coniferous tree stands. Forest cover in the catchments was between 0% and 9% for the four northernmost lakes and Stor Björsjön, but substantially higher for the more easterly situated, lower‐elevation lakes Louvvajaure and Dunnervattnet (58% and 59%, respectively) (Table 2). This potential error should, however, contribute to a systematic underestimation, due to the saturation effect of NDVI, i.e., the phenomenon that NDVI shows little or no change at higher vegetation density (Gao et al. 2023) and thus does not accurately reflect the development of understory vegetation. Because this potential error would be more likely to occur in forests where spatial heterogeneity is higher (Baloloy et al. 2018), we expect the share of birch forest in the catchment to some degree contribute to an underestimation of greenness. Shippert et al. (1995, Figure 5) identified a NDVI of 0.7 as a threshold above which saturation may become problematic for plant biomass estimation. Our sites, however, had no or only single NDVI_max_ values exceeding 0.7, with the exception of Dunnervattnet, which also had the largest share of forest in the catchment among our study lakes. Furthermore, the gradual, long‐term decline in summer snow patches could have contributed to an increase in NDVI, especially for the lakes at the highest latitudes and elevations (e.g., Latnjajaure, Njalakjaure). The fact that our sequestration estimates of N‐ and P‐sequestration are based on aboveground biomass of green vegetation only further emphasizes the conservative nature of these estimates. This is because NDVI measures green biomass, which is likely accompanied by increases in biomass of aboveground structural plant parts as well as belowground root systems. The review by Iversen et al. (2015) emphasizes that the average ratio of belowground to aboveground biomass for different functional tundra plant types varies between 2.7 and 8.2, further reinforcing our conclusion.

Changes in grazing pressure may also affect vegetation cover and NDVI values, and the Sami People use these high‐elevation landscapes for reindeer herding during the Arctic summer. Our observed increase in vegetation development could, however, not be explained by declines in grazing pressure as the reindeer population in the Swedish part of the Scandes has been relatively stable since 2004 (Sami Parliament 2024). Reindeer populations declined by 13% (from 253,200 to 219,300 individuals) during 1995–2001, and then rapidly recovered to a relatively stable population of 250,846 ± 6024 individuals during 2004–2020 (interannual mean ± SD, data not shown). These numbers should, however, be seen as underestimates, as they are based on winter counts after the annual slaughter, whereas summer populations of reindeer are ca. 50%–60% larger, after calving in spring. These populations have been stable since 2004, and our data thus show that vegetation development occurred despite an increase in reindeer population size in the late 1990s. Although grazing by lemmings may also impact vegetation cover during years with peak populations (Olofsson et al. 2012), this likely did not impact our results. Lemming populations in the Scandes peaked for the first time in many years in 2011 (Olofsson et al. 2012), but effects on the catchment‐scale NDVI_max_ were not apparent in our time series (Figures 5 and S3).

Warming‐induced greening of landscapes is likely to be further stimulated by the expected increase in wet (i.e., pluvial) precipitation and increased weathering and decomposition of organic matter (nutrient regeneration) in soils (Hartley et al. 1999) as the climate warms. Increases in wet precipitation could potentially contribute to the dilution of run‐off (Isles et al. 2023), but this explanation was rebutted by Huser et al. (2018) and Nilsson et al. (2024) for Swedish lakes and rivers, respectively. Increased warming could, however, contribute to increased sorption of P to recalcitrant soil P fractions such as iron oxyhydroxides and/or clays, as shown by Tian et al. (2023) for forested sites in the Austrian Alps. In addition, while slight increases in soil pH can affect P sorption in soils, the increased formation of P‐binding metals (e.g., iron‐ and aluminum‐oxyhydroxides) may limit P mobility in the drainage area (Huser et al. 2018). This has been particularly addressed in studies on the recovery from acidification (Kopáček et al. 2015). However, our study lakes are situated in a region that has been minimally affected by acid deposition in the 1960s–1980s, so this would have contributed only marginally to the declining Total‐P water concentrations in the current study. In contrast, we infer that the sequestration of Total‐P by drainage area vegetation is by far the predominant process that explains the loss of Total‐P in run‐off and ultimately lake Total‐P concentrations.

### 4.1 Consequences of Oligotrophication for Lake Food Webs

The loss of nutrients in run‐off appears to push these clearwater lakes to ultra‐oligotrophic conditions with possible negative effects on whole‐lake productivity. However, analyses of decadal monitoring data on phytoplankton biovolume and chlorophyll *a* show no significant temporal trends for these lakes (W. Goedkoop, unpublished data). This may be attributed to the high share of mixotrophs that typically predominate the phytoplankton communities of oligotrophic clearwater lakes (Bergström et al. 2003; Waibel et al. 2019). Mixotrophs have the ability to alternate between photosynthesis and phagotrophy on bacteria and organic particles, which is advantageous in these ultraoligotrophic lakes where dark conditions predominate during the long winter. While planktonic primary producers are strongly dependent on nutrient water concentrations, benthic algae, that predominate primary production in oligotrophic clearwater lakes (Vadeboncoeur et al. 2003), may be less affected by declining surface water nutrient concentrations because they can obtain nutrients from the sediment (Vadeboncoeur et al. 2006). Therefore, climate‐driven trends including higher water temperatures, deeper stratification, and a longer ice‐free season in these subarctic lakes may instead have a stimulatory effect on primary production and overall lake productivity. However, nutrient‐ and warming‐induced changes in the assemblage composition of benthic primary producers toward a larger share of cyanobacteria, which are favored by low‐nutrient conditions and warming (Schartau et al. 2022), will result in lower basal resource quality, reduced trophic transfer efficiency, and a drop in overall ecosystem productivity (Brett and Müller‐Navarra 1997).

## 5 Conclusions/Outlook

Our unique time series of nutrient water concentrations and vegetation monitoring data, together with satellite imagery, revealed landscape‐level consequences of ongoing climate warming. These data also contribute to addressing the challenge of linking satellite‐based greenness to field‐level observations (Piao et al. 2019). We demonstrate strong links between terrestrial vegetation development, i.e., landscape greening, and the ongoing loss of nutrients (primarily P) from lakes. These changes, together with longer ice‐free periods and warmer waters, will likely alter the productivity and composition of basal resources in these lakes and may have yet unforeseen consequences for their unique biodiversity (Heino et al. 2020), including Arctic char (Muhlfeld et al. 2024; Kangosjärvi et al. 2024). Due to the lack of monitoring in many parts of the remote Arctic region (Goedkoop et al. 2022), however, such ecological changes are seldom observed or the underlying processes identified. While increased vegetation development (i.e., increased CO_2_ uptake) may imply negative feedback on climate change, warming‐induced oligotrophication may have negative effects on freshwater ecosystem health and services, including fisheries production, that are of key importance to the Indigenous Peoples and other residents of the Arctic.

## Author Contributions

**Willem Goedkoop:** conceptualization, data curation, formal analysis, funding acquisition, investigation, project administration, resources, visualization, writing – original draft, writing – review and editing. **Sven Adler:** conceptualization, data curation, formal analysis, investigation, methodology, resources, software, validation, writing – original draft. **Brian Huser:** data curation, investigation, methodology, writing – review and editing. **Hans Gardfjell:** data curation, validation, writing – review and editing. **Danny C. P. Lau:** conceptualization, investigation, validation, writing – review and editing.

## Conflicts of Interest

The authors declare no conflicts of interest.

## Supporting information


Data S1.

## Data Availability

The data that support the findings of this study are openly available in the Swedish National Data Service (SND) at https://doi.org/10.5878/ytfc‐gk90. Water chemistry data are openly available from the MVM Soil, Water and Environmental data platform at https://miljodata.slu.se/MVM/Search. Temperature data were obtained from Climate Research Unit Time‐series database (CRU TS version 4.06) at https://crudata.uea.ac.uk/cru/data/hrg/. NDVI data were extracted from the NOAA Climate data record (normalized NDVI version 4) using the Google Earth engine. https://www.ncei.noaa.gov/products/climate‐data‐records and are available at https://doi.org/10.7289/V5PZ56R6. Data on reindeer population size have been extracted from the Swedish Sami parliament https://www.sametinget.se/statistik/renhjorden.
