# Supplementary material for: Climate Change‐Induced Landscape Alterations Increase Nutrient Sequestration and Cause Severe Oligotrophication of Subarctic Lakes
Source: Glob Chang Biol. 2025 Jul 9;31(7):e70314. doi: 10.1111/gcb.70314 (PMC12238918; doi:10.1111/gcb.70314)
Supplement: Supplementary file 1 — Data S1. [file GCB-31-e70314-s001.docx]

Goedkoop et al. Supplementary materials

Figure S1. Temporal trends (1985–2020) in monthly mean air temperature (June–September) for nine study lakes. Line fits show a simple GAM smooth fit. Sen’s slopes and p-values (Mann-Kendall) are given in the panels.


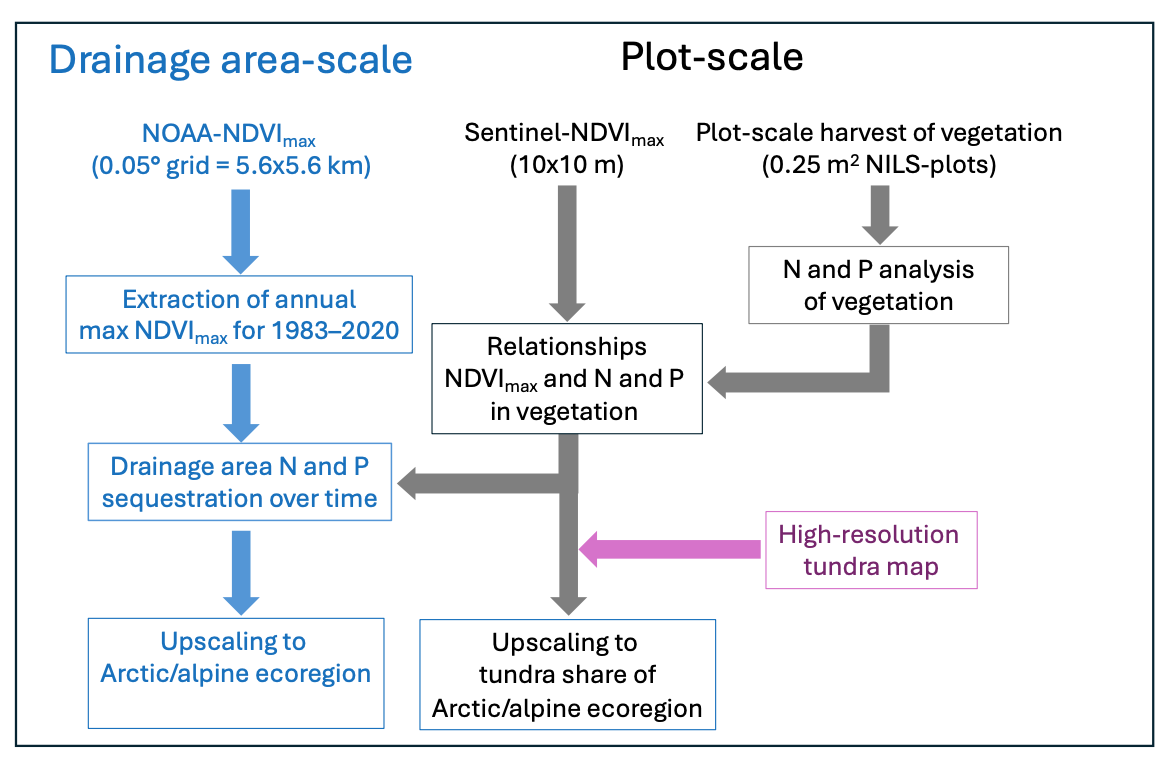


Figure S2. Flow chart illustrating the procedure by which satellite data of different resolution, i.e., covering both the catchment scale (blue) and plot-scale (grey), were used to upscale plot-scale data on nutrient concentrations in vegetation to drainage area sequestration of nutrients. For further explanation see text.

Figure S3. Temporal trends (1983–2020) in NDVI_max_ for the drainage areas of the nine study lakes. Line fits show a simple GAM smooth fit. Sen’s slopes and p-values (Mann-Kendall) are given in the panels.
